# Implication of different frailty criteria in older people with atrial fibrillation: a prospective cohort study

**DOI:** 10.1186/s12877-023-04330-1

**Published:** 2023-09-27

**Authors:** Junpeng Liu, Ke Chai, Wanrong Zhu, Minghui DU, Chen Meng, Lin Yang, Lingling Cui, Di Guo, Ning Sun, Hua Wang, Jiefu Yang

**Affiliations:** 1 Department of Cardiology, Beijing Hospital, National Center of Gerontology, Institute of Geriatric Medicine, Chinese Academy of Medical Sciences, No.1, Da Hua Road, Dongcheng District, Beijing, 100730 People’s Republic of China; 2 Peking University Fifth School of Clinical Medicine, Beijing, 100730 China

**Keywords:** Frailty, Atrial fibrillation, Prognosis

## Abstract

**Background:**

the prevalence of physical and multidimensional frailty and their prognostic impact on clinical outcomes in patients with atrial fibrillation (AF) is unclear.

**Objective:**

to evaluated frailty in a cohort of patients with AF according to different criteria, and studied the prevalence and its prognostic impact on clinical outcomes.

**Methods:**

in this multicenter prospective cohort, 197 inpatients ≥ 65 years old with AF were recruited from September 2018 to April 2019.We used Fried Frailty phenotype (Fried) to assess physical frailty, and comprehensive geriatric assessment-frailty index (CGA-FI) to assess multidimensional frailty. The primary outcome was a composite of all-cause mortality or rehospitalization.

**Results:**

the prevalence of frailty was determined as 34.5% by Fried, 42.6% by CGA-FI. Malnutrition and ≥ 7 medications were independently associated with frailty. Kaplan-Meier survival curve showed that the presence of frailty by CGA-FI had significantly lower all-cause mortality or rehospitalization survival rate (log-rank P = 0.04) within 1 year. Multivariate Cox regression adjusted for age and sex showed that the frailty by CGA-FI was significantly associated with the risk of all-cause mortality or rehospitalization within 1 year (HR 1.79, 95% CI 1.10–2.90). However, those associations were absent with the physical frailty. After broader multivariate adjustment, those associations were no longer statistically significant for both types of frailty.

**Conclusions:**

in older people with AF, Multidimensional frailty is more significantly associated with a composite of all-cause mortality or rehospitalization within 1 year than physical frailty, but these association are attenuated after multivariate adjustment.

**Clinical trial registration:**

ChiCTR1800017204; date of registration: 07/18/2018.

**Supplementary Information:**

The online version contains supplementary material available at 10.1186/s12877-023-04330-1.

## Key points

The prevalence of multidimensional frailty (42.6% by CGA-FI) was higher than that of physical frailty (34.5% by Fried) in older people with AF.

Malnutrition and polypharmacy were independent risk factors for frailty.

In older people with AF, multidimensional frailty was more significantly associated with a composite of all-cause mortality or rehospitalization within 1 year than physical frailty, but these association were attenuated after multivariate adjustment.

**Supplementary Information:**

The online version contains supplementary material available at 10.1186/s12877-023-04330-1.

## Introduction

As the global population continues to age, atrial fibrillation (AF) has become one of the top medical and social concerns worldwide [1]. This increasing burden is a challenge for health systems worldwide. Frailty is a common geriatric syndrome characterized by a state of increased vulnerability to endogenous and exogenous stressors, due to age-related declines in physiologic reserve and function across multiple physiologic systems [2–4]. Previous studies have revealed that frailty is a common comorbid condition in patients with AF [5–7].

There are various proposed definitions and conceptual frameworks of frailty. They could be mainly divided into two categories: (1) Physical frailty: frailty was defined as a physical syndrome, of which the Fried Frailty phenotype (Fried) is the most widely researched [3]. It is a biological model of frailty, including weak grip strength, exhaustion, unintentional weight loss, low physical activity and slow walking. (2) Multidimensional frailty: frailty was defined as a state of vulnerability resulting from accumulation of health deficits. The Comprehensive geriatric assessment-frailty index (CGA-FI) proposed by Rockwood and based on which the suitable FI could be created according to the characteristics of different populations [8] was used as a major tool.

Few studies have evaluated frailty association with clinical outcomes in patients with AF. To the best of our knowledge, no study has ever simultaneously evaluated different tools to quantify frailty and its prognostic impact on clinical outcomes in the same cohort of patients with AF. Therefore, we evaluated frailty in a cohort of patients with AF according to different criteria, and studied the prevalence of frailty and its prognostic impact on clinical outcomes.

## Methods

### Data and participants

**Fig. 1:**
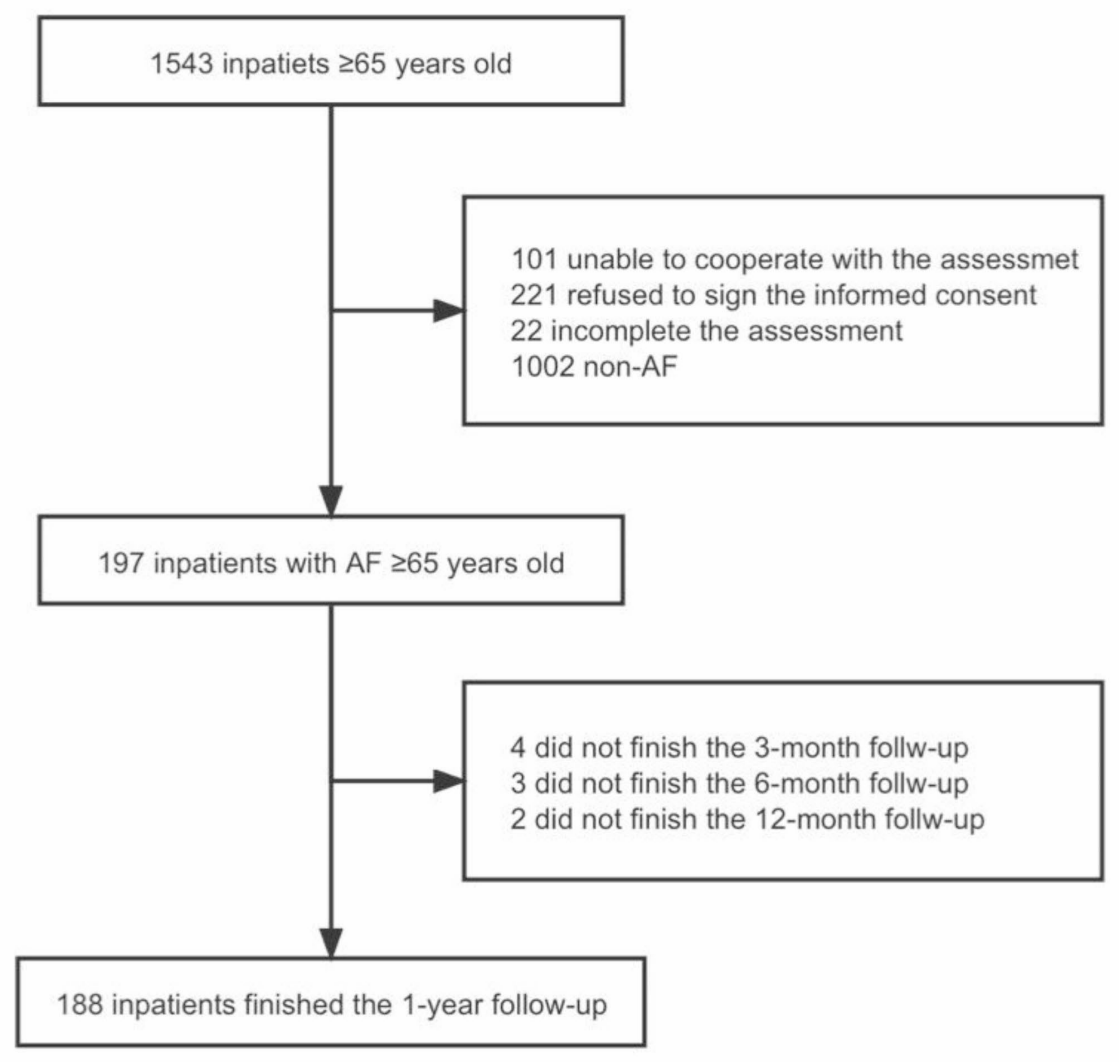
Flow chart of the study cohort

We used data from a prospective observational cohort study on frailty in China (Trial registration: ChiCTR1800017204; date of registration: 07/18/2018) .The Study recruited ≥ 65years older people who were consecutively admitted to 3 tertiary referral hospitals in Beijing, China, from September 2018 to April 2019, which approved by the Ethics Committee of Beijing Hospital (approval no. 2018BJYYEC-121-02). For the current analyses, we included patients with a diagnosis of current or resolved atrial fibrillation (Fig. 1).

### Information collection

Information was gleaned by fixed investigators, who had passed the survey training test, through a case report form to ensure the validity of the collected data. Data were collected and managed through Research Electronic Data Capture (REDCap) and the entire study was supervised by Peking University Clinical Research Institute. Baseline data collection included sociodemographics, hospitalization information, medical history, comorbidities, physical examinations, laboratory tests, echocardiographic data and medications.

### Frailty assessment

We used 2 different frailty tools to assess the frailty of hospitalized older people with AF.

1. Fried Frailty phenotype (Fried).

The Fried frailty phenotype was commonly used to assess frailty consisting of 5 criteria: unintentional weight loss, self-reported exhaustion, low grip strength, slow walking speed, and low physical activity [3]. The scores were between 0 and 5. Patients with a score ≥ 3 were classified as frailty. The detailed descriptions were presented in Supplementary Table 1.

2. The comprehensive geriatric assessment-frailty index (CGA-FI).

The CGA-FI proposed by Rockwood and based on which the suitable FI could be created according to the characteristics of different populations was used [8]. According to the core criteria, we selected 48 variables to construct the CGA-FI, including activities of daily living, chronic disease, depression, anxiety, loneliness, Mini-Mental State Examination (MMSE) [9], geriatric syndrome, insomnia, body mass index, calf circumference, peak flow, grip strength, and 4-m walking speed. Patients with a score ≥ 0.25 were classified as frailty. The detail cut-off values were in Supplementary Table 2.

### Comorbidities

Comorbidities were measured by the Charlson comorbidity index (CCI) [10](see Supplementary Table 3). Patients consented to the use of electronic medical records to identify previous clinical history of hypertension, cardiac artery disease, myocardial infarction, heart failure, peripheral vascular disease, diabetes and cerebrovascular disease. We used the Chinese version of the mini-mental state examination (MMSE) [9] and clock drawing test (CDT) [11] to define cognitive impairment: (1) below 24 points of MMSE or (2) 24 ≤ MMSE ≤ 26 and incorrect CDT. Malnutrition was defined according to the short form mini nutritional assessment (MNA-SF) ≤ 7 points [12].

### Study outcomes and follow-up

The primary outcome of this study was a composite of all-cause mortality or rehospitalization. All events were independently reviewed. Clinical follow-up was routinely performed via clinical visit and/or telephone interview at 3, 6 and 12 months.

### Statistical analysis

Patients were categorized into 2 groups based on the presence of frialty by 2 different criteria (Fried and CGA-FI). Continuous data expressed as the mean standard deviation (SD), and independent *t* test was performed for intergroup comparison. Non-normally distributed variables were reported as median (25th -75th percentile), and Wilcoxon rank-sum test was performed for intergroup comparison. The categorical variables, presented as counts and percentages, were compared using the Chi-squared test or Fisher’s exact test. Venn diagrams were used to illustrate the relationship between frailty assessment tools.

Independent predictors of frailty according to different tool were determined in a multivariable logistic regression model. The Logistic regression was performed using the enter method, in which all independent variables were entered into the logistic regression, which include demographic, clinical, laboratorial, and echocardiagraphic variables.

Cumulative survival curves were estimated by Kaplan-Meier methods and compared between groups using the log-rank test. To determine the independent association between all-cause mortality or rehospitalization and frail according to different tool, multivariable Cox proportional hazard regression model was used to examine the associations of frailty with risk of all-cause mortality or rehospitalization. Model 1 adjusted for age and sex. Model 2 adjusted for the same factors as the multivariable logistic regression models. The Cox models were tested for the proportional hazards assumption and linearity of continuous variables. We checked using the variance inflation factor ensuring that the variance inflation factor for each variable was < 10. All statistical tests were bilateral tests, and a *P* value of < 0.05 was considered to indicate statistical significance. All the analyses were performed using SAS software, version 9.4 (SAS Institute Inc).

## Results

A total of 197 consecutive older people with AF were studied, including 158 patients (80.2%) from cardiovascular wards (Fig. 1). There were 57.4% males (113/197) in the study and the average age was 77.5 ± 7.1 years. Among them, 103 cases (52.3%) were paroxysmal AF and 94 cases (47.7%) were persistent AF, the CHA_2_DS_2_-VASc score was 4.4 ± 1.6, 91 cases (47.7%) were ≥ 5, the HAS-BLED score was 1.9 ± 0.7, 36 cases (18.3%) were ≥ 3. Common comorbidities included hypertension (75.6%), coronary heart disease (48.7%), diabetes (29.4%), stroke /TIA (23.4%) and heart failure (23.9%).

### Prevalence of frailty and baseline characteristics

**Fig. 2:**
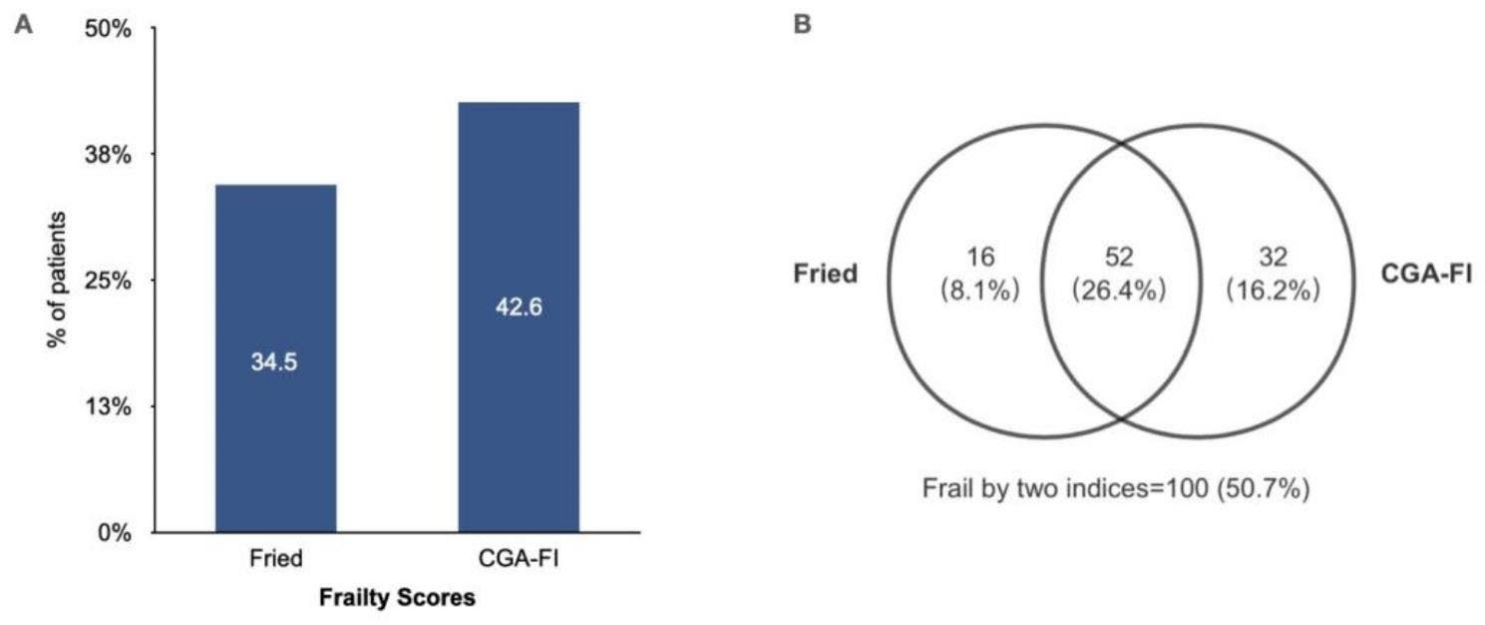
Prevalence of frailty by Fried and CGA-FI in AF cohort. A, Bar graph showing prevalence of frailty by Fried and CGA-FI in AF cohort; B, Venn diagrams showing the relationship between Fried and CGA-FI in detecting frailty in patients with AF. Fried = Fried frailty phenotype; CGA-FI = comprehensive geriatric assessment-frailty index

The prevalence of frailty in older people with AF was determined as 34.5% by Fried, 42.6% by CGA-FI. 26.4% (N = 52) of patients were classified as frail by all 2 assessment tools (Fig. 2).

The baseline characteristics of patients according to the presence of frailty by different criteria are summarized in Table 1. Regardless of the criteria, frail patient with AF was older, more female, had higher CHA_2_DS_2_-VAS_C_ and Charison Comorbidity Index score, more had heart failure, cognitive impairment, and malnutrition, had higher D-dimer and NT-proBNP, had larger left atrial diameter, took more oral medications, and had fewer interventional or surgical procedures.

**Table 1:** Baseline characteristics According to the presence of Frailty by Fried and CGA-FI in patients with AF

	Fried			CGA-FI		
	Non-frail	Frail		Non-frail	Frail	
	(n = 129)	(n = 68)	p Value	(n = 113)	(n = 84)	p Value
Demographics						
Age, y	76.0 ± 6.8	80.3 ± 6.6	**< 0.001**	74.9 ± 6.5	80.9 ± 6.4	**< 0.001**
Male	82 (63.6)	31 (45.6)	**0.02**	74 (65.5)	39 (46.4)	**0.007**
University or higher	58 (45.0)	20 (29.4)	**0.002**	52 (46.0)	26 (31.0)	0.07
Living alone	10 (7.8)	5 (7.4)	0.92	8 (7.1)	7 (8.3)	0.74
HR, bpm	77.0 ± 19.3	77.0 ± 19.1	0.99	77.2 ± 20.0	76.7 ± 18.2	0.86
SBP, mmHg	131.2 ± 18.5	126.4 ± 17.9	0.08	131.1 ± 16.8	127.5 ± 20.3	0.18
DBP, mmHg	74.8 ± 11.4	73.6 ± 10.9	0.51	75.0 ± 11.0	73.5 ± 11.5	0.34
BMI, kg/m2	25.1 ± 3.4	24.0 ± 3.7	0.07	25.0 ± 3.2	24.5 ± 4.0	0.38
Grip Strength, kg	27.5 ± 8.3	17.6 ± 6.5	**< 0.001**	27.9 ± 8.6	19.1 ± 6.9	**< 0.001**
Gait Speed, s	5.3 (4.6,6.8)	8.4 (6.5,10.2)	**< 0.001**	5.4 (4.5,6.6)	8.3 (5.7,10.2)	**< 0.001**
AF						
Praxysmal AF	68 (52.7)	35 (51.5)	0.87	64 (56.6)	39 (46.4)	0.16
CHA_2_DS_2_-VASc			**< 0.001**			**< 0.001**
≤ 4	84 (65.1)	22 (32.4)		83 (73.5)	23 (27.4)	
≥ 5	45 (34.9)	46 (67.6)		30 (26.5)	61 (72.6)	
HAS-BLED			0.54			**0.001**
≤ 2	107 (82.9)	54 (79.4)		101 (89.4)	60 (71.4)	
≥ 3	22 (17.1)	14 (20.6)		12 (10.6)	24 (28.6)	
Comorbidities						
CCI	1.0 (1.0,2.0)	2.0 (1.0,3.0)	**0.004**	1.0 (1.0,2.0)	2.0 (2.0,4.0)	**< 0.001**
HTN	99 (76.7)	50 (73.5)	0.62	80 (70.8)	69 (82.1)	0.07
CAD	59 (45.7)	37 (54.4)	0.25	50 (44.2)	46 (54.8)	0.14
MI	11 (8.5)	9 (13.2)	0.3	7 (6.2)	13 (15.5)	**0.03**
HF	25 (19.4)	22 (32.4)	**0.04**	13 (11.5)	34 (40.5)	**< 0.001**
PVD	21 (16.3)	13 (19.1)	0.62	13 (11.5)	21 (25.0)	**0.01**
Diabetes	40 (31.0)	18 (26.5)	0.51	31 (27.4)	27 (32.1)	0.47
Stroke/TIA	29 (22.5)	17 (25.0)	0.69	22 (19.5)	24 (28.6)	0.14
CI	28 (21.7)	29 (42.6)	**0.002**	13 (11.5)	44 (52.4)	**< 0.001**
Malnutrition	2 (2.3)	6 (8.8)	**0.04**	1 (0.9)	8 (9.5)	**0.004**
Laboratory						
Hb, g/l	130.8 ± 20.4	125.5 ± 19.4	0.08	133.6 ± 18.5	122.7 ± 20.6	**< 0.001**
Alb, g/l	39.4 ± 2.9	38.6 ± 3.7	0.13	39.6 ± 2.8	38.5 ± 3.7	**0.02**
D-dimer, ng/ml	139.5 (91.0,230.0)	208.0 (97.0,380)	**0.03**	125.0 (82.0,200.0)	208.0 (114.0,432.0)	**< 0.001**
hsCRP, mg/dl	1,1 (0.8,2.3)	1.1 (0.7,2.6)	0.83	1.0 (0.7,1.8)	1.4 (0.8,3.9)	**0.05**
NT-proBNP, pg/ml	608 (152.5–1526.0)	1009.1 (354.4-2214.5)	**0.01**	569.3 (154.8–1355.0)	1300.0 (334.9–2252.0)	**< 0.001**
Echocardiaography						
LVEF, %	59.1 ± 8.4	57.7 ± 9.4	0.34	60.1 ± 7.9	56.7 ± 9.5	**0.009**
LAD, mm	40.7 ± 7.5	44.5 ± 7.8	**0.002**	40.4 ± 7.6	44.2 ± 7.5	**< 0.001**
Treatment						
OAC	61 (47.3)	29 (42.6)	0.74	54 (47.8)	36 (42.9)	0.53
≥ 7 medications	49 (38.0)	40 (58.8)	**0.005**	35 (31.0)	54 (64.3)	**< 0.001**
Intervention/surgery	61 (47.3)	21 (30.9)	**0.03**	64 (56.6)	18 (21.4)	**< 0.001**

Values are presented as mean ± SD or median ( interquartile range: 25th to 75th

percentiles) or number (percentage)

AF = atrial fibrillation; Alb = albumin; BMI = body mass index; CAD = coronary artery disease; CCI = charison comorbidity Index; CI = Cognitive impairment; DBP = diastolic blood pressure; Hb = hemoglobin; HF = heart failure; HR = heart rate; hs-CRP = high-sensitive C-reactive protein; HTN = hypertension; LAD = left atrial anteroposterior diameter; LVEF = left ventricular ejection fraction; MI = myocardial infarction; NT-proBNP = N-terminal pro-B-type natriuretic peptide; OAC = oral anticoagulants; PVD = peripheral vascular disease; SBP = systolic blood pressure; TIA = transient ischemic attack

### Independent predictors of frailty by different criteria

The results of univariate and multivariate analyses for identifying the presence of frailty according to each criterion in older people with AF are shown in Supplementary Tables 4–5. Regardless of the criteria, Malnutrition and ≥ 7 medications were independently associated with frailty in patients with AF.

### Clinical outcome

During the 1-year follow-up period, 4 patients were lost to follow-up at 3 months, 3 at 6 months, and 2 at 1 year. 188 patients completed 1-year follow-up, with a total loss rate of 4.6% (Fig. 1). The primary end point event (all-cause mortality or rehospitalization) occurred in 82 patients (41.6%) .

**Fig. 3:**
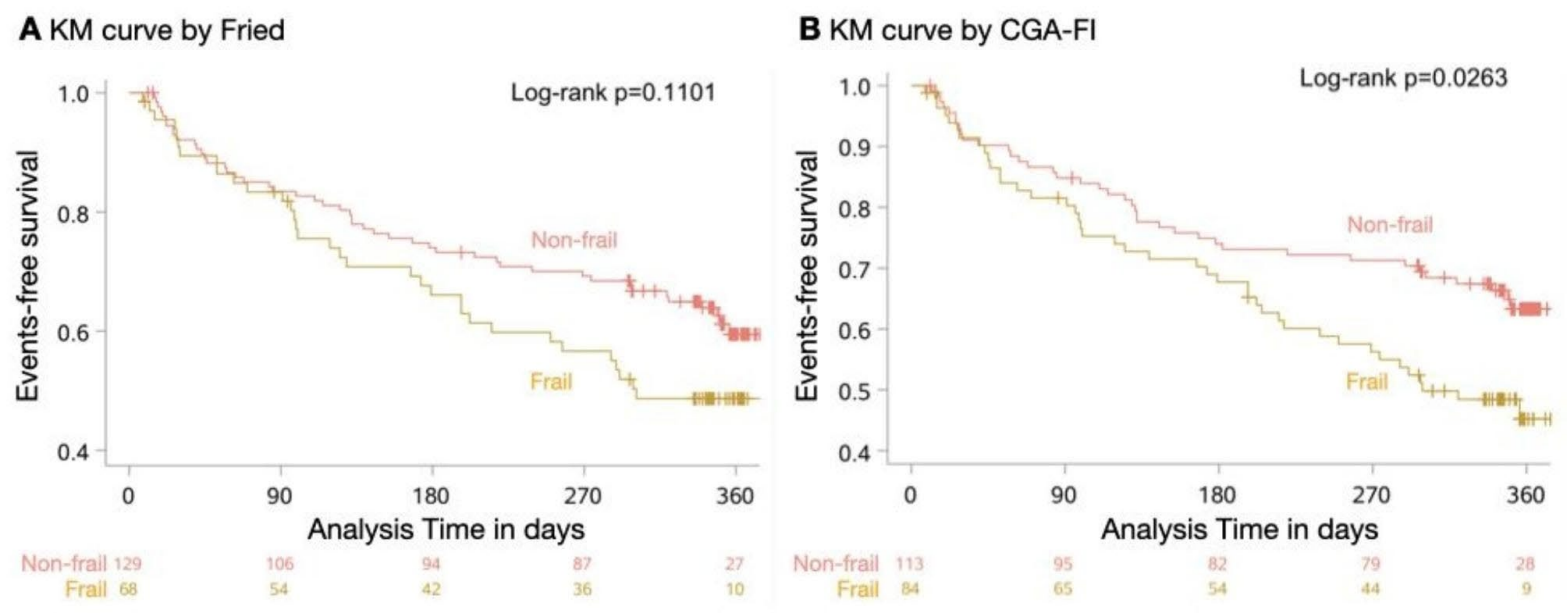
Kaplan-Meier Analysis of all-cause mortality or rehospitalization Events According to the presence of Frailty by Fried (A) and CGA-FI (B) in patients with AF

Kaplan-Meier survival curve showed that the presence of frailty by CGA-FI had significantly lower all-cause mortality or rehospitalization survival rate (log-rank P = 0.04) within 1 year, but not according to Fried (log-rank P = 0.11) (Fig. 3).

Table 2 shows the results for serially adjusted models. In Model 1, which adjusted for age and gender, the frailty by CGA-FI was significantly associated with the risk of all-cause mortality or rehospitalization within 1 year in older people with AF (HR 1.79, 95%CI 1.10–2.90, P = 0.02). However, this association was absent with the Fried criteria (HR 1.49, 95%CI 0.92–2.42, P = 0.10). In Model 2, which adjusted for age, sex, education, living alone, smoking, drinking, BMI, CHA2DS2-VASc ≥ 5, HAS-BLED ≥ 3, CCI, HF, CI, Malnutrition, HR, Log Hb, Log Alb, Log hsCRP, Log NT-proBNP, LAD, LVEF, ≥ 7 medications, those associations were no longer statistically significant for both criteria. Detailed results are provided in Supplementary Tables 6–7.

**Table 2:** Univariate and Multivariate Analyses for the Primary Outcome of all-cause mortality or rehospitalization

				Multivariable Analysis					
	Univariable Analysis			Model 1			Model 2		
	*HR*	*95%CI*	*P value*	*HR*	*95%CI*	*P value*	*HR*	*95%CI*	*P value*
Age	1.12	0.83–1.51	0.45						
Male	1.24	0.80–1.93	0.34						
Frailty by Fried	1.43	0.92–2.22	0.11	1.49	0.92–2.42	0.10	1.60	0.86–2.96	0.14
Frailty by CGA-FI	1.63	1.06–2.51	0.03	1.79	1.10–2.90	0.02	1.74	0.94–3.24	0.08

Abbreviations as in Table 1

Model 1 adjusted for age and sex;

Model 2 adjusted for age, sex, education, living alone, smoking, drinking, BMI, CHA_2_DS_2_-VASc ≥ 5, HAS-BLED ≥ 3, CCI, HF, CI, Malnutrition, HR, Log Hb, Log Alb, Log hsCRP, Log NT-proBNP, LAD, LVEF, ≥ 7 medications

## Discussion

In this study, we used a prospective, cohort of consecutive individual with AF to evaluate the association between clinical outcome and the presence of frailty as determined by 2 of the most commonly used assessment tools–Fried and CGA-FI. The main findings were as follows: (1) In patients with AF, The prevalence of multidimensional frailty (42.6% by CGA-FI) was higher than that of physical frailty (34.5% by Fried) ; (2) In patients with AF, the presence of frailty was associated with older, more female, higher CHA_2_DS_2_-VAS_C_ and CCI score, more heart failure, cognitive impairment, and malnutrition, higher D-dimer and NT-proBNP, larger left atrial diameter, taking more oral medications, and fewer interventional or surgical procedures regardless of the criteria. (3) Regardless of the criteria used, Malnutrition and ≥ 7 medications were independent risk factors for frailty in patients with AF. (4) AF patients with multidimensional frailty had significantly higher all-cause mortality or rehospitalization rate within 1 year, but not with physical frailty. (5) After adjusting for age and gender, only the presence of multidimensional frailty by CGA-FI was significantly associated with the risk of all-cause mortality or rehospitalization within 1 year in older people with AF. Such significant association was not observed with the presence of physical frailty by Fried. After broader multivariate adjustment, those associations were no longer statistically significant for both types of frailty.

Frailty is a common geriatric syndrome characterized by a state of increased vulnerability to endogenous and exogenous stressors, due to age-related declines in physiologic reserve and function across multiple physiologic systems [13]. Previous studies have revealed that frailty is a common comorbid condition in patients with AF and the prevalence in AF is higher than in the general population [5–7]. However, The reported incidence of frailty in patients with AF is highly variable, ranging from 5.9–89.5% [6, 7, 14–17], which was influenced by many factors, such as age, study population, the evaluating instruments, et al. Overall, the prevalence of frailty in hospital AF patients is significantly higher than that in community AF patients, and the higher the age of enrolment, the higher the frailty prevalence. Frailty tool is an important factor affecting the incidence of frailty in AF. Our study found that the prevalence of multidimensional frailty in patient with AF according to CGA-FI is higher than physical frailty according to Fried, which is consistent with the general population [18]. However, in the heart failure population, physical frailty is more prominent [19].

Risk factors for the frailty in span a wide range of aspects and conditions, covering sociodemographic, clinical, biological domains and lifestyle-related [20]. Similarly, the risk factors associated with frailty according to different criteria were different. Mlynarska et al [14] used Tilbrug frailty indicator (TFI) [21] to assess multidimensional frailty and reported that age and the EHRA score were important predictors of multidimensional frailty syndrome in patients with AF. Our findings are also consistent with theirs and found age and comorbidity were associated with multidimensional frailty in patients with AF. Further, we identified that age and comorbidity was not as closely associated with physical frailty as with multidimensional frailty in patients with AF. In addition, Malnutrition and ≥ 7 medications were independent risk factors for frailty in patients with AF regardless of the types of frailty. As an independent risk factor for the onset of frailty, there is a large amount of evidence for malnutrition in the general population [18, 22] and different diseases populations, such as coronary heart disease [23] and heart failure [24]. Moreover, nutritional therapy is also one of the important measures of frailty intervention [25, 26]. Interestingly, we found polypharmacy was independently associated with frailty in AF. This finding is rarely reported in the previous literature. Polypharmacy means more comorbidities. Previous studies showed that frailty and multimorbidity may contribute to each other [27]. Our study found that in patient with AF, multimorbidity was associate with multidimensional frailty, but not with physical frailty. Therefore, there may be other factors that contribute to the effect of polypharmacy on frailty, such as drug interactions and side effects.

AF associated with frailty has been shown previously to have worse outcome [28–30]. In a multicenter cohort of AF patients study, Madhavan et al [15] showed frailty was associated with increased risk of death (HR1.29, 95% CI 1.08–1.55, p = 0.006). They used the American Geriatric Society’s Geriatric Evaluation and Management Tool to assess frailty, which was just as the Fried. Wikinson et al [6] showed in those with AF and eligible for OAC, frailty was associated with increased risk of death (HR for severe frailty compared with fit 4.09, 95% CI 3.43–4.89, p < 0.05). Frailty was estimated using the eFI (Electronic frailty index) [31], in which the proportion of deficits (symptoms and signs, abnormal laboratory values, disability or disease state) from 36 possible deficits was calculated. As a result, both physical frailty and multidimensional frailty are associate with poor prognosis in patients with AF. However, there is currently no study examining the prognostic role of physical frailty and multidimensional frailty simultaneously in the same cohort of patients with AF. In our study, we found that multidimensional frailty associated with increased risk of all-cause mortality or rehospitalization within 1 year in older people with AF, and in the same cohort of patients with AF, we simultaneously evaluated physical frailty and its prognostic impact on clinical outcomes. Such significant association was not observed with the presence of physical frailty. These results suggest that the frailty index, which encompasses multidimensional impairments, is more suitable for assessing frailty in people with AF. Additionally, more attention should be given to comprehensive chronic disease management for older individuals with AF to reduce mortality and rehospitalization rates. Moreover, these associations were attenuated after multivariate adjustment For both types of frailty. This suggests that associations between frailty and risk of all-cause mortality or rehospitalization in patients with AF may be attributable to other patients’ prognostic factors.

Although frailty and AF are frequent comorbidities and share common risk factors, the direction and strength of the association of frailty with AF onset, subsequent disease incidence, and mortality are not completely understood. In a study of 2053 participants in the FHS (Framingham Heart Study), Orkaby et al [7] sought to examine both the association between frailty and incident AF and the association between prevalent AF and frailty status. Frailty was defined using the Fried phenotype. They did not find a statistically meaningful relationship between AF and frailty. The findings may be limited by sample size. In addition, we should note that this only represents the relationship between physical frailty and AF, and whether multidimensional frailty is the case is uncertain.

Our study also has important limitations. First, although our study is a multi-center study, the sample size is relatively limited, and the cases are limited to individual in tertiary hospitals, which needs to be verified in different levels of medical institutions and communities. Second, although the multivariate Cox regression model was used in control for various potential confounders, residual or unknown confounders were still unavoidable. Furthermore, the follow-up time of our study is only one year, and future studies with larger sample size and longer follow-up are needed.

## Conclusions

In summary, among older people with AF, we found that the prevalence of multidimensional frailty was higher than that of physical frailty. Malnutrition and polypharmacy were independent risk factors for frailty in patients with AF regardless of the criteria. Multidimensional frailty was more significantly associated with a composite of all-cause mortality or rehospitalization within 1 year than physical frailty, but these association were attenuated after multivariate adjustment.

### Electronic supplementary material

Below is the link to the electronic supplementary material.

Supplementary Material 1

Supplementary Material 2

## Data Availability

The datasets used and/or analyzed during the current study are available from the corresponding author on reasonable request.

## Abbreviations

AF: Atrial fibrillation
BMI: Body mass index
CCI: Charlson comorbidity index
CDT: Clock drawing test
CGA-FI: Comprehensive geriatric assessment-frailty index
eFI: Electronic frailty index
FHS: Framingham Heart Study
Fried: Fried Frailty phenotype
hsCRP: High sensitivity C-reactive protein
LAD: Left atrial diameter
LVEF: Left ventricular ejection fraction
MMSE: Mini-mental state examination
NT-proBNP: N-terminal pro-brain natriuretic peptide

## References

[1] Schnabel RB, Yin X, Gona P (2015). 50 year trends in atrial fibrillation prevalence, incidence, risk factors, and mortality in the framingham heart study: a cohort study. Lancet.

[2] Rockwood K, Stadnyk K, MacKnight C, McDowell I, Hebert R, Hogan DB (1999). A brief clinical instrument to classify frailty in elderly people. Lancet.

[3] Fried LP, Tangen CM, Walston J (2001). Frailty in older adults: evidence for a phenotype. J Gerontol a Biol Sci Med Sci.

[4] Clegg A, Young J, Iliffe S, Rikkert MO, Rockwood K (2013). Frailty in elderly people. Lancet.

[5] Polidoro A, Stefanelli F, Ciacciarelli M, Pacelli A, Di Sanzo D, Alessandri C (2013). Frailty in patients affected by atrial fibrillation. Arch Gerontol Geriatr.

[6] Wilkinson C, Clegg A, Todd O (2021). Atrial fibrillation and oral anticoagulation in older people with frailty: a nationwide primary care electronic health records cohort study. Age Ageing.

[7] Orkaby AR, Kornej J, Lubitz SA (2021). Association between frailty and atrial fibrillation in older adults: the framingham heart study offspring cohort. J Am Heart Assoc.

[8] Searle SD, Mitnitski A, Gahbauer EA, Gill TM, Rockwood K (2008). A standard procedure for creating a frailty index. Bmc Geriatr.

[9] Folstein MF, Folstein SE, McHugh PR (1975). Mini-mental state. A practical method for grading the cognitive state of patients for the clinician. J Psychiatr Res.

[10] Charlson ME, Pompei P, Ales KL, MacKenzie CR (1987). A new method of classifying prognostic comorbidity in longitudinal studies: development and validation. J Chronic Dis.

[11] Yao SM, Zheng PP, Liang YD (2020). Predicting non-elective hospital readmission or death using a composite assessment of cognitive and physical frailty in elderly inpatients with cardiovascular disease. Bmc Geriatr.

[12] Rubenstein LZ, Harker JO, Salva A, Guigoz Y, Vellas B (2001). Screening for undernutrition in geriatric practice: developing the short-form mini-nutritional assessment (mna-sf). J Gerontol a Biol Sci Med Sci.

[13] Hoogendijk EO, Afilalo J, Ensrud KE, Kowal P, Onder G, Fried LP (2019). Frailty: implications for clinical practice and public health. Lancet.

[14] Mlynarska A, Mlynarski R, Golba KS (2017). Older age and a higher ehra score allow higher levels of frailty syndrome to be predicted in patients with atrial fibrillation. Aging Male.

[15] Madhavan M, Holmes DN, Piccini JP (2019). Association of frailty and cognitive impairment with benefits of oral anticoagulation in patients with atrial fibrillation. Am Heart J.

[16] Mlynarska A, Mlynarski R, Marcisz C, Golba KS (2020). Modified frailty as a novel factor in predicting the maintenance of the sinus rhythm after electrical cardioversion of atrial fibrillation in the elderly population. Clin Interv Aging.

[17] Wilkinson C, Wu J, Searle SD (2020). Clinical outcomes in patients with atrial fibrillation and frailty: insights from the engage af-timi 48 trial. Bmc Med.

[18] Liang YD, Zhang YN, Li YM (2019). Identification of frailty and its risk factors in elderly hospitalized patients from different wards: a cross-sectional study in china. Clin Interv Aging.

[19] Sze S, Pellicori P, Zhang J, Weston J, Clark AL (2019). Identification of frailty in chronic heart failure. Jacc Heart Fail.

[20] Clegg A, Bates C, Young J (2018). Development and validation of an electronic frailty index using routine primary care electronic health record data. Age Ageing.

[21] Gobbens RJ, van Assen MA, Luijkx KG, Wijnen-Sponselee MT, Schols JM (2010). The tilburg frailty indicator: psychometric properties. J Am Med Dir Assoc.

[22] Hong X, Yan J, Xu L, Shen S, Zeng X, Chen L (2019). Relationship between nutritional status and frailty in hospitalized older patients. Clin Interv Aging.

[23] Lyu H, Wang C, Jiang H, Wang P, Cui J (2021). Prevalence and determinants of frailty in older adult patients with chronic coronary syndrome: a cross-sectional study. Bmc Geriatr.

[24] Yaku H, Kato T, Morimoto T (2020). Risk factors and clinical outcomes of functional decline during hospitalisation in very old patients with acute decompensated heart failure: an observational study. Bmj Open.

[25] Ngandu T, Lehtisalo J, Solomon A (2015). A 2 year multidomain intervention of diet, exercise, cognitive training, and vascular risk monitoring versus control to prevent cognitive decline in at-risk elderly people (finger): a randomised controlled trial. Lancet.

[26] Lana A, Rodriguez-Artalejo F, Lopez-Garcia E (2015). Dairy consumption and risk of frailty in older adults: a prospective cohort study. J Am Geriatr Soc.

[27] Vetrano DL, Palmer K, Marengoni A (2019). Frailty and multimorbidity: a systematic review and meta-analysis. J Gerontol a Biol Sci Med Sci.

[28] Wilkinson C, Todd O, Clegg A, Gale CP, Hall M (2019). Management of atrial fibrillation for older people with frailty: a systematic review and meta-analysis. Age Ageing.

[29] Nguyen TN, Cumming RG, Hilmer SN (2016). Atrial fibrillation in older inpatients: are there any differences in clinical characteristics and pharmacological treatment between the frail and the non-frail?. Intern Med J.

[30] Perera V, Bajorek BV, Matthews S, Hilmer SN (2009). The impact of frailty on the utilisation of antithrombotic therapy in older patients with atrial fibrillation. Age Ageing.

[31] Clegg A, Bates C, Young J (2016). Development and validation of an electronic frailty index using routine primary care electronic health record data. Age Ageing.

