# Supplementary material for: Implication of different frailty criteria in older people with atrial fibrillation: a prospective cohort study
Source: BMC Geriatr. 2023 Sep 27;23:604. doi: 10.1186/s12877-023-04330-1 (PMC10537815; doi:10.1186/s12877-023-04330-1)
Supplement: Supplementary file 1 — Supplementary Material 1 [file 12877_2023_4330_MOESM1_ESM.docx]

**Supplemental Material :**

**Supplementary** **Table S1.** Evaluation of frailty by Fried criteria.

**Supplementary** **Table S2.** Factors of CGA-FI.

**Supplementary** **Table S3.** Charlson Co-morbidity Index.

**Supplementary** **Table S4.** Risk factor associated with Frailty by Fried in patients with AF by Univariable and Multivariable Logistic Regression.

**Supplementary** **Table S5.** Risk factor associated with Frailty by CGA-FI in patients with AF by Univariable and Multivariable Logistic Regression

**Supplementary** **Table S6.** Univariate and Multivariate Cox regression (Model 2) analyses of risk factors associated with the Primary Outcome of all-cause death or rehospitalization within 1 year.
