# Supplementary material for: Implication of different frailty criteria in older people with atrial fibrillation: a prospective cohort study
Source: BMC Geriatr. 2023 Sep 27;23:604. doi: 10.1186/s12877-023-04330-1 (PMC10537815; doi:10.1186/s12877-023-04330-1)
Supplement: Supplementary file 2 — Supplementary Material 2 [file 12877_2023_4330_MOESM2_ESM.docx]

**Supplemental Tables**

**Supplementary Table S1.** Evaluation of frailty by Fried criteria

|  | **Male** | **Female** |
| --- | --- | --- |
| Weight loss | >4.5kg over past year or >5% of previous year’s body weight | |
| Exhaustion | Any question ≥3 days | |
|  | 1. How often in the last week did you feel like everything you did was an effort? | |
|  | 2. How often in the last week did you feel like you cannot get going? | |
| Grip strength decline | Grip strength (Kg) criterion for frailty | |
|  | BMI≤24kg/m^2^，≤29kg | BMI≤23kg/m^2^，≤17kg |
|  | 24<BMI≤28kg/m^2^，≤30kg | 23<BMI≤26kg/m^2^，≤17.3kg |
|  | BMI＞28kg/m^2^，≤32kg | 26＜BMI≤29kg/m^2^，≤18kg |
|  |  | BMI＞29kg/m^2^，≤21kg |
| Walk Time | Time to walk 5 metre criterion for frailty | |
|  | Height≤173cm，≥6.1s | Height≤159cm，≥6.1s |
|  | Height＞173cm，≥5.3s | Height＞159cm，≥5.3s |
| Physical Activity | Used the short version of the Minnesota Leisure Time Activity questionnaire | |
|  | <383kcal/week (walking 2.5h/week) | <270kcal/week (walking 2h/week) |

Abbreviations: BMI=body mass index

**Supplementary Table S2 .** Factors of CGA-FI

| Frailty index | Cut-off |
| --- | --- |
| 1.Need help bathing | Yes=1, No=0 |
| 2. Need help dressing | Yes=1, No=0 |
| 3. Need help using the toilet | Yes=1, No=0 |
| 4. Need help getting in / out of chair | Yes=1, No=0 |
| 5. Need help feeding | Yes=1, No=0 |
| 6. Incontinence | Yes=1, No=0 |
| 7. Need help shopping | Yes=1, No=0 |
| 8. Need help with finances | Yes=1, No=0 |
| 9. Need help using transportation | Yes=1, No=0 |
| 10. Need help using telephone | Yes=1, No=0 |
| 11. Need help managing medications | Yes=1, No=0 |
| 12. Need help with housekeeping | Yes=1, No=0 |
| 13. Need help preparing meals | Yes=1, No=0 |
| 14. Need help washing clothes | Yes=1, No=0 |
| 15. Hypertension | Yes=1, No=0 |
| 16. Coronary heart disease | Yes=1, No=0 |
| 17. Atrial fibrillation | Yes=1, No=0 |
| 18. Congestive heart failure | Yes=1, No=0 |
| 19. Peripheral artery disease | Yes=1, No=0 |
| 20. Chronic Lung disease | Yes=1, No=0 |
| 21. Sleep apnea hypopnea syndrome | Yes=1, No=0 |
| 22. Diabetes | Yes=1, No=0 |
| 23. Thyroid dysfunction | Yes=1, No=0 |
| 24. Peptic ulcer | Yes=1, No=0 |
| 25. Stroke | Yes=1, No=0 |
| 26. Chronic kidney disease | Yes=1, No=0 |
| 27. Osteoarthritis | Yes=1, No=0 |
| 28. Osteoporosis | Yes=1, No=0 |
| 29. Parkinson’s disease | Yes=1, No=0 |
| 30. Cancer | Yes=1, No=0 |
| 31. Depression | Yes=1(GDS-5items≥2), No=0(GDS-5items<2) |
| 32. Anxiety | Yes=1(HADS-A≥8), No=0(HADS-A<8) |
| 33. Loneliness | Yes=1, No=0 |
| 34. Cognition | MMSE<18 =1; 18≤MMSE<24 =0.5; MMSE≥ 24 =0 |
| 35. Visual impairment | Yes=1, No=0 |
| 36. Hearing impairment | Yes=1, No=0 |
| 37. Chewing impairment | Yes=1, No=0 |
| 38. Fall history | Yes=1, No=0 |
| 39. Chronic constipation | Yes=1, No=0 |
| 40. Chronic pain | Yes=1, No=0 |
| 41. Insomnia | Yes=1(AIS≥6), No=0(AIS<6) |
| 42. Depend on assistive devices | Yes=1, No=0 |
| 43. Take exercise outside | Yes=1, No=0 |
| 44. Body mass index (BMI) (kg/m^2^) | BMI≤18 or BMI≥28 =1; 25≤BMI<28 =0.5; 18<BMI<25 =0 |
| 45. Calf circumference (cm) | ≤25^th^ percentile =1;  25^th^ to 50^th^ percentiles =0.5;  >50^th^ percentile =0 |
| 46. Peak flow (liters/min) |  |
| 47. Grip strength (kg) |  |
| 48. 4m-walking speed (m/s) |  |

Abbreviations: AIS=Athens Insomnia Scale; CGA-FI=comprehensive geriatric assessment-frailty index; GDS-5items=5-item Geriatric Depression Scale; HADS-A=Hospital Anxiety and Depression Scale-Anxiety; MMSE=Mini-Mental State Examination; .

**Supplementary Table S3.** Charlson Co-morbidity Index

| **All Charlson conditions** | **Weights** |
| --- | --- |
| Myocardial infarction | 1 |
| Congestive heart failure | 1 |
| Peripheral vascular disease | 1 |
| Cerebral vascular accident | 1 |
| Dementia | 1 |
| Chronic pulmonary disease | 1 |
| Connective tissue disease | 1 |
| Ulcer disease | 1 |
| Mild liver disease | 1 |
| Diabetes mellitus | 1 |
| Diabetes mellitus with chronic complications | 2 |
| Hemiplegia, paraplegia | 2 |
| Moderate/severe renal disease | 2 |
| Any tumor | 2 |
| Leukemia | 2 |
| Lymphoma | 2 |
| Metastatic solid tumor | 3 |
| Moderate/severe liver disease | 3 |
| AIDS | 6 |

Abbreviations: AIDS=Acquired Immune Deficiency Syndrome.

**Supplementary Table S4.** Risk factor associated with Frailty by Fried in patients with AF by Univariable and Multivariable Logistic Regression

| Variables | Univariable Analysis | | | Multivaribale Analysis | | |
| --- | --- | --- | --- | --- | --- | --- |
|  | *OR* | *95%CI* | *P value* | *OR* | *95%CI* | *P value* |
| Age | 2.54 | 1.60-4.04 | <0.001 | 1.54 | 0.77-3.08 | 0.22 |
| Male | 0.48 | 0.26-0.87 | 0.02 | 0.86 | 0.29-2.54 | 0.79 |
| University or higher | 0.23 | 0.10-0.54 | <0.001 | 0.13 | 0.04-0.48 | **0.002** |
| Living alone | 0.94 | 0.31-2.88 | 0.92 | 0.46 | 0.10-2.02 | 0.30 |
| Smoking | 0.36 | 0.18-0.73 | 0.004 | 0.55 | 0.16-1.89 | 0.34 |
| Drinking | 0.22 | 0.10-0.48 | <0.001 | 0.30 | 0.09-0.99 | **0.05** |
| BMI | 0.93 | 0.86-1.02 | 0.11 | 0.95 | 0.85-1.07 | 0.41 |
| CHA_2_DS_2_-VASc≥5 | 3.90 | 2.09-7.28 | <0.001 | 3.09 | 1.17-8.20 | **0.02** |
| HAS-BLED≥3 | 1.26 | 0.60-2.66 | 0.54 | 0.43 | 0.13-1.39 | 0.16 |
| CCI | 1.26 | 1.05-1.51 | 0.01 | 1.25 | 0.89-1.75 | 0.19 |
| HF | 1.99 | 1.02-3.89 | 0.04 | 0.37 | 0.11-1.19 | 0.10 |
| CI | 2.68 | 1.42-5.07 | 0.002 | 0.54 | 0.19-1.54 | 0.25 |
| Malnutrition | 4.06 | 0.98-16.80 | 0.05 | 15.73 | 1.70-145.48 | **0.02** |
| HR | 1.00 | 0.98-1.02 | 0.99 | 0.99 | 0.97-1.01 | 0.43 |
| Log Hb | 0.32 | 0.06-1.60 | 0.16 | 3.28 | 0.35-30.63 | 0.30 |
| Log Alb | 0.05 | 0.00-1.48 | 0.08 | 0.41 | 0.00-56.36 | 0.72 |
| Log hsCRP | 1.10 | 0.87-1.40 | 0.42 | 0.88 | 0.61-1.27 | 0.48 |
| Log D-dimer | 1.40 | 1.02-1.93 | 0.04 | 1.11 | 0.73-1.68 | 0.63 |
| Log NT-proBNP | 1.36 | 1.07-1.72 | 0.01 | 1.15 | 0.79-1.68 | 0.45 |
| LAD | 1.07 | 1.02-1.11 | 0.002 | 1.06 | 1.00-1.12 | **0.03** |
| LVEF | 0.98 | 0.95-1.02 | 0.35 | 1.02 | 0.97-1.08 | 0.39 |
| ≥7 medications | 2.33 | 1.28-4.25 | 0.006 | 2.94 | 1.25-6.90 | **0.01** |

Abbreviations as in Table 1.

**Supplementary Table S5.** Risk factor associated with Frailty by CGA-FI in patients with AF by Univariable and Multivariable Logistic Regression

| Variables | Univariable Analysis | | | Multivaribale Analysis | | |
| --- | --- | --- | --- | --- | --- | --- |
|  | *OR* | *95%CI* | *P value* | *OR* | *95%CI* | *P value* |
| Age | 3.97 | 2.42-6.51 | <0.001 | 6.19 | 2.40-15.96 | **<0.001** |
| Male | 0.46 | 0.26-0.81 | 0.008 | 0.66 | 0.17-2.53 | 0.54 |
| University or higher | 0.38 | 0.17-0.85 | 0.02 | 2.07 | 0.41-10.52 | 0.38 |
| Living alone | 1.19 | 0.41-3.43 | 0.74 | 0.57 | 0.12-2.71 | 0.48 |
| Smoking | 0.49 | 0.26-0.92 | 0.03 | 0.51 | 0.11-2.39 | 0.40 |
| Drinking | 0.27 | 0.14-0.54 | <0.001 | 0.44 | 0.10-1.96 | 0.28 |
| BMI | 0.96 | 0.89-1.04 | 0.36 | 1.08 | 0.94-1.25 | 0.29 |
| CHA_2_DS_2_-VASc≥5 | 7.34 | 3.89-13.86 | <0.001 | 1.86 | 0.56-6.13 | 0.31 |
| HAS-BLED≥3 | 3.37 | 1.57-7.22 | 0.0002 | 1.87 | 0.46-7.65 | 0.38 |
| CCI | 1.98 | 1.55-2.53 | 0.01 | 1.72 | 1.02-2.89 | **0.04** |
| HF | 5.23 | 2.54-10.79 | <0.001 | 0.57 | 0.12-2.62 | 0.47 |
| CI | 8.46 | 4.12-17.37 | <0.001 | 8.57 | 2.29-32.03 | **0.001** |
| Malnutrition | 11.79 | 1.44-96.19 | 0.02 | 68.18 | 3.07-1514.1 | **0.008** |
| HR | 1.00 | 0.98-1.01 | 0.86 | 0.97 | 0.94-1.00 | 0.06 |
| Log Hb | 0.03 | 0.00-0.24 | <0.001 | 0.14 | 0.00-5.01 | 0.28 |
| Log Alb | 0.02 | 0.00-0.50 | 0.02 | 2.74 | 0.00-1674.7 | 0.76 |
| Log hsCRP | 1.46 | 1.12-1.89 | 0.005 | 1.12 | 0.68-1.83 | 0.66 |
| Log D-dimer | 1.84 | 1.31-2.59 | <0.001 | 1.46 | 0.85-2.49 | 0.17 |
| Log NT-proBNP | 1.54 | 1.21-1.96 | <0.001 | 1.02 | 0.62-1.65 | 0.95 |
| LAD | 1.07 | 1.03-1.11 | 0.002 | 1.05 | 0.98-1.12 | 0.20 |
| LVEF | 0.96 | 0.92-0.99 | 0.01 | 0.98 | 0.91-1.06 | 0.62 |
| ≥7 medications | 4.01 | 2.20-7.30 | <0.001 | 3.72 | 1.29-10.69 | **0.01** |

Abbreviations as in Table 1.

**Supplementary Table S6.** Univariate and Multivariate Cox regression (Model 2) analyses of risk factors associated with the Primary Outcome of all-cause death or rehospitalization within 1 year.

| Variables | Univariable Analysis | | | Multivaribale Analysis | | |
| --- | --- | --- | --- | --- | --- | --- |
|  | *HR* | *95%CI* | *P value* | *HR* | *95%CI* | *P value* |
| Age | 1.12 | 0.83-1.51 | 0.45 | 0.83 | 0.55-1.25 | 0.37 |
| Male | 1.24 | 0.80-1.93 | 0.34 | 1.87 | 0.87-4.02 | 0.11 |
| Frailty (Fried) | 1.43 | 0.92-2.22 | 0.11 | 1.60 | 0.86-2.96 | 0.14 |
| University or higher | 0.90 | 0.49-1.65 | 0.74 | 1.10 | 0.41-2.99 | 0.85 |
| Living alone | 1.02 | 0.42-2.49 | 0.96 | 1.47 | 0.59-3.61 | 0.41 |
| Smoking | 1.10 | 0.70-1.74 | 0.68 | 0.85 | 0.44-1.66 | 0.64 |
| Drinking | 1.08 | 0.67-1.73 | 0.76 | 1.41 | 0.68-2.94 | 0.36 |
| BMI | 0.96 | 0.80-1.02 | 0.16 | 0.99 | 0.91-1.07 | 0.73 |
| CHA_2_DS_2_-VASc≥5 | 1.52 | 0.99-2.33 | 0.06 | 1.18 | 0.62-2.23 | 0.62 |
| HAS-BLED≥3 | 1.24 | 0.75-2.07 | 0.41 | 0.97 | 0.47-2.02 | 0.95 |
| CCI | 1.29 | 1.13-1.47 | < 0.01 | 1.23 | 1.00-1.52 | 0.05 |
| HF | 1.84 | 1.17-2.92 | 0.009 | 1.19 | 0.58-2.46 | 0.63 |
| CI | 1.13 | 0.71-1.81 | 0.61 | 1.22 | 0.57-2.60 | 0.61 |
| Malnutrition | 1.19 | 0.36-3.92 | 0.78 | 0.50 | 0.14-1.75 | 0.28 |
| HR | 1.00 | 0.99-1.02 | 0.37 | 1.01 | 0.99-1.03 | 0.23 |
| Log Hb | 0.30 | 0.13-0.69 | 0.004 | 0.24 | 0.06-1.02 | 0.05 |
| Log Alb | 0.06 | 0.00-1.09 | 0.06 | 0.09 | 0.00-3.87 | 0.21 |
| Log hsCRP | 1.09 | 0.93-1.29 | 0.29 | 0.94 | 0.76-1.17 | 0.59 |
| Log D-dimer | 1.19 | 0.96-1.47 | 0.11 | 0.97 | 0.71-1.32 | 0.85 |
| Log NT-proBNP | 1.13 | 0.97-1.32 | 0.12 | 1.06 | 0.87-1.30 | 0.57 |
| LAD | 1.03 | 1.00-1.06 | 0.09 | 1.02 | 0.98-1.06 | 0.32 |
| LVEF | 0.98 | 0.95-1.00 | 0.04 | 1.00 | 0.97-1.04 | 0.75 |
| ≥7 medications | 1.06 | 0.69-1.62 | 0.79 | 0.55 | 0.30-1.01 | 0.06 |

Abbreviations: AF=atrial fibrillation; Alb=albumin; BMI=body mass index; CAD=coronary artery disease; CCI=charison comorbidity Index; CI=Cognitive impairment; Hb=hemoglobin; HF=heart failure; HR=heart rate; hs-CRP=high-sensitive C-reactive protein; LAD=left atrial anteroposterior diameter; LVEF=left ventricular ejection fraction; NT-proBNP=N-terminal pro-B-type natriuretic peptide

Model 2 adjusted for age, sex, education, living alone, smoking, drinking, BMI, CHA_2_DS_2_-VASc≥5, HAS-BLED≥3, CCI, HF, CI, Malnutrition, HR, Log Hb, Log Alb, Log hsCRP, Log NT-proBNP, LAD, LVEF, ≥7 medications

**Supplementary Table S7. Univariate and Multivariate Cox regression (Model 2) analyses of risk factors associated with the Primary Outcome of all-cause death or rehospitalization within 1 year.**

| Variables | Univariable Analysis | | | Multivaribale Analysis | | |
| --- | --- | --- | --- | --- | --- | --- |
|  | *HR* | *95%CI* | *P value* | *HR* | *95%CI* | *P value* |
| Age | 1.12 | 0.83-1.51 | 0.45 | 0.75 | 0.49-1.15 | 0.19 |
| Male | 1.24 | 0.80-1.93 | 0.34 | 1.90 | 0.91-3.98 | 0.09 |
| Frailty (CGA-FI) | 1.63 | 1.06-2.51 | 0.03 | 1.74 | 0.94-3.24 | 0.08 |
| University or higher | 0.90 | 0.49-1.65 | 0.74 | 0.86 | 0.33-2.25 | 0.76 |
| Living alone | 1.02 | 0.42-2.49 | 0.96 | 1.40 | 0.57-3.46 | 0.47 |
| Smoking | 1.10 | 0.70-1.74 | 0.68 | 0.78 | 0.39-1.56 | 0.48 |
| Drinking | 1.08 | 0.67-1.73 | 0.76 | 1.38 | 0.66-2.87 | 0.39 |
| BMI | 0.96 | 0.80-1.02 | 0.16 | 0.98 | 0.91-1.06 | 0.60 |
| CHA_2_DS_2_-VASc≥5 | 1.52 | 0.99-2.33 | 0.06 | 1.17 | 0.61-2.23 | 0.64 |
| HAS-BLED≥3 | 1.24 | 0.75-2.07 | 0.41 | 0.92 | 0.44-1.91 | 0.82 |
| CCI | 1.29 | 1.13-1.47 | < 0.01 | 1.25 | 1.01-1.55 | 0.04 |
| HF | 1.84 | 1.17-2.92 | 0.009 | 1.10 | 0.55-2.22 | 0.78 |
| CI | 1.13 | 0.71-1.81 | 0.61 | 0.98 | 0.46-2.12 | 0.96 |
| Malnutrition | 1.19 | 0.36-3.92 | 0.78 | 0.50 | 0.14-1.74 | 0.27 |
| HR | 1.00 | 0.99-1.02 | 0.37 | 1.01 | 1.00-1.03 | 0.18 |
| Log Hb | 0.30 | 0.13-0.69 | 0.004 | 0.29 | 0.07-1.14 | 0.08 |
| Log Alb | 0.06 | 0.00-1.09 | 0.06 | 0.10 | 0.00-3.78 | 0.21 |
| Log hsCRP | 1.09 | 0.93-1.29 | 0.29 | 0.93 | 0.75-1.16 | 0.52 |
| Log D-dimer | 1.19 | 0.96-1.47 | 0.11 | 0.94 | 0.70-1.28 | 0.72 |
| Log NT-proBNP | 1.13 | 0.97-1.32 | 0.12 | 1.08 | 0.87-1.33 | 0.49 |
| LAD | 1.03 | 1.00-1.06 | 0.09 | 1.02 | 0.98-1.06 | 0.29 |
| LVEF | 0.98 | 0.95-1.00 | 0.04 | 1.01 | 0.98-1.04 | 0.59 |
| ≥7 medications | 1.06 | 0.69-1.62 | 0.79 | 0.53 | 0.29-0.99 | 0.04 |

Abbreviations as in Table S4.

Model 2 adjusted for age, sex, education, living alone, smoking, drinking, BMI, CHA_2_DS_2_-VASc≥5, HAS-BLED≥3, CCI, HF, CI, Malnutrition, HR, Log Hb, Log Alb, Log hsCRP, Log NT-proBNP, LAD, LVEF, ≥7 medications
